# The Global Dam Watch database of river barrier and reservoir information for large-scale applications

**DOI:** 10.1038/s41597-024-03752-9

**Published:** 2024-10-08

**Authors:** Bernhard Lehner, Penny Beames, Mark Mulligan, Christiane Zarfl, Luca De Felice, Arnout van Soesbergen, Michele Thieme, Carlos Garcia de Leaniz, Mira Anand, Barbara Belletti, Kate A. Brauman, Stephanie R. Januchowski-Hartley, Kimberly Lyon, Lisa Mandle, Nick Mazany-Wright, Mathis L. Messager, Tamlin Pavelsky, Jean-François Pekel, Jida Wang, Qingke Wen, Marcus Wishart, Tianqi Xing, Xiao Yang, Jonathan Higgins

**Affiliations:** 1 Department of Geography, McGill University, Montreal, QC H3A 0B9 Canada; 2 Global Water Security Center, University of Alabama, Tuscaloosa, AL 35487 USA; 3 Department of Geography, King’s College London, London, WC2B 4BG UK; 4 Department of Geoscience, Eberhard Karls University of Tübingen, 72076 Tübingen, Germany; 5 European Commission, Joint Research Centre (JRC), 21027 Ispra, Italy; 6 UN Environment Programme - World Conservation Monitoring Centre (UNEP-WCMC), Cambridge, CB3 0DL UK; 7 World Wildlife Fund, Washington, DC 20037 USA; 8 Department of BioSciences, Swansea University, Swansea, SA2 8PP UK; 9 CNRS-EVS, University Jean-Monnet, University of Lyon, Saint-Etienne, 42023 France; 10 Water Global Practice, World Bank, Washington, DC 20433 USA; 11 Natural Capital Project, Stanford University, Stanford, CA 94305 USA; 12 Canadian Wildlife Federation, Kanata, ON K2M 2W1 Canada; 13 RiverLy Research Unit, National Research Institute for Agriculture, Food and Environment (INRAE), Villeurbanne, 69100 France; 14 Department of Earth, Marine and Environmental Sciences, University of North Carolina, Chapel Hill, NC 27514 USA; 15 Department of Geography and Geographic Information Science, University of Illinois Urbana-Champaign, Urbana, IL 61801 USA; 16 Department of Geography and Geospatial Sciences, Kansas State University, Manhattan, KS 66506 USA; 17 Aerospace Information Research Institute, Chinese Academy of Sciences, Beijing, 100101 China; 18 Department of Earth Sciences, Southern Methodist University, Dallas, TX 75205 USA; 19 Global Freshwater Team, The Nature Conservancy, Chicago, IL 60611 USA

**Keywords:** Hydrology, Environmental sciences

## Abstract

There are millions of river barriers worldwide, ranging from wooden locks to concrete dams, many of which form associated impoundments to store water in small ponds or large reservoirs. Besides their benefits, there is growing recognition of important environmental and social trade-offs related to these artificial structures. However, global datasets describing their characteristics and geographical distribution are often biased towards particular regions or specific applications, such as hydropower dams affecting fish migration, and are thus not globally consistent. Here, we present a new river barrier and reservoir database developed by the Global Dam Watch (GDW) consortium that integrates, harmonizes, and augments existing global datasets to support large-scale analyses. Data curation involved extensive quality control processes to create a single, globally consistent data repository of instream barriers and reservoirs that are co-registered to a digital river network. Version 1.0 of the GDW database contains 41,145 barrier locations and 35,295 associated reservoir polygons representing a cumulative storage capacity of 7,420 km^3^ and an artificial terrestrial surface water area of 304,600 km^2^.

## Background & Summary

Human societies have altered rivers for millennia. Across the world, barrier structures serving a wide variety of purposes have been built to store, divert, or regulate flows in order to either harness the benefits of water resources (e.g., water supply, irrigation, hydropower generation, navigation) or prevent harmful events (e.g., flood protection). However, the proliferation of instream barriers built over the past century is unprecedented. These barriers include a wide spectrum of types, sizes, and functional designs, ranging from regulation weirs on small creeks to shipping locks across some of the largest rivers in the world and tall concrete dams that can store multiple years’ worth of river flow in their associated reservoirs (see Table 1). While built to provide services to humans, mostly related to economic growth and development, these structures can cause unintended and often complex ecological and societal consequences, ranging from modified aquatic habitats and natural flow regimes to human health implications^1–5^. Even when only considering the largest ~20,000 dams, the majority of large rivers worldwide are fragmented, with free-flowing rivers mostly confined to remote regions of the Arctic, the Amazon Basin, and, to a lesser extent, the Congo Basin^6^. The number of smaller barriers is much higher with more than 1 million barriers fragmenting Europe’s rivers alone^7^.

**Table 1:** Terminology for different types of river barriers and reservoirs as commonly used, including in this study.

Term	General definition or usage
**Barrier**	Used here as a catch-all term for infrastructure that sits across all or part of a river or stream bed, i.e., a longitudinal barrier.
**Dam**	A solid barrier that can be made of earth, wood, metal, rock, concrete, or a combination of materials that is built across a river to impound and manage its flow. Dams typically create reservoirs but can also be run-of-river structures. Many types of dams exist, including main dams that expand beyond the river into the adjacent floodplain, or subsidiary (saddle) dams constructed at low points along the perimeter of the reservoir. In some regions, the term *dam* is used interchangeably with *reservoir*.
**Weir**	A barrier built across the width of a river to alter the river’s flow characteristics. Other purposes of weirs can include sediment regulation and riverbed stabilization. Weirs generally allow water to flow over the crest, but they can cause temporary or limited ponding effects. These structures can also be referred to as low-head dams.
**Barrage**	A barrier built in a river to divert water into a channel typically for navigation or irrigation purposes.
**Lock**	A device used for raising and lowering boats and other watercraft between stretches of a river of differing levels. These structures typically only create small and temporary reservoirs.
**Reservoir**	Used here as a catch-all term for the artificial waterbody formed behind an instream barrier or through other means of storing water (such as by excavation or diversion into a depression). Reservoirs can range from small ponds to lake-size waterbodies and can be permanent or fluctuate in their water volume and extent. They may also include *regulated lakes*.
**Impoundment**	Can be used synonymously with *reservoir*, but often refers to more specific types of impounded reservoirs such as tailings ponds, water-filled mining pits, or wastewater lagoons.
**Regulated lake**	Natural lake whose water level can be regulated by some form of controlling structure. Its storage capacity typically refers only to the controlled part of the entire water volume. Examples include lakes Baikal, Victoria, and Superior.

Adapted and expanded from Belletti *et al*.^7^ and Garcia de Leaniz & O’Hanley^47^. Terms and definitions are not meant to be mutually exclusive and may have varying regional interpretations.

Given their importance, existing river barrier and reservoir datasets at full global extent (see Table 2) have facilitated a multitude of studies to assess the individual and cumulative effects of instream barriers and impounded waters on people, ecosystems, and river dynamics across the world (e.g., refs. ^6,8–12^). But despite many iterations of dataset improvements and the increased inclusion of novel remote sensing imagery and machine learning techniques, each of the existing datasets has its own challenges and limitations. These shortcomings are typically a reflection of the different goals, intentions, or data sources used in the dataset creation. For example, constraints may be due to a focus on large dams or certain types of dams only; records may be missing precise location information; and spatial coverage may be skewed due to data gaps in some regions and duplicates in others. In particular, all contemporary global datasets fail to capture smaller barriers on small- to mid-sized rivers, yet these can have outsized cumulative effects^13–17^. In addition, some of the datasets are not freely available for general use.

**Table 2:** Contemporary, fully global river barrier and reservoir datasets, including the GDW database, and their main characteristics.

Dataset name and *reference*	Number and type of records	Focus	Number/type of attributes	Versioning and license
**World Register of Dams (WRD)** *ICOLD*^45^	58,713 dams*	Dams >15 m high, or 3–15 m with a reservoir greater than 0.03 km^3^	>40 attributes, incl. height, purpose, year, volume	Annual updates Paid subscription fee
**FAO AQUASTAT Database on Dams** *FAO*^48^	>14,000 dams	Medium to large dams; records were partially included in GRanD	>25 attributes, incl. height, purpose, year, volume	Last update in 2014 Free (non-commercial)
**GRanD** (v1.4) *Lehner et al*.^27^	7,424 dams 7,378 reservoirs	Large reservoirs (volume ≥0.1 km^3^) and their dams, incl. attributes	>50 attributes, incl. height, purpose, year, volume, discharge	Sporadic updates Free (non-commercial)
**GOODD** *Mulligan et al*.^33^	38,667 dams	Medium to large dams >150 m long and with reservoirs >500 m long	Only the point locations of barriers are provided	Sporadic updates Free (non-commercial)
**GROD** (v1.1) *Yang et al*.^34^	30,549 barriers	Multiple types of barriers on rivers wider than 30 m	Barrier class, incl. dam, lock, low-head dam	No updates pending Free (CC BY 4.0)
**FHRED** *Zarfl et al*.^35^	>3,700 dams	Proposed hydropower dams with nominal capacity ≥1 MW	River name, nominal capacity (MW), proposed year	Sporadic updates Free (non-commercial)
**GeoDAR** (v1.1) *Wang et al*.^46^	24,783 dams 21,515 reservoirs	Georeferenced location of dams from WRD and their reservoirs	Attributes from GRanD (v1.3) included, from WRD excluded	Sporadic updates Free (CC BY 4.0)
**Global Water Watch (GWW)** *Donchyts et al*.^49^	71,208 reservoirs^†^	Reservoirs ≥10 ha derived from multi-annual multi-sensor satellite data	Surface water area changes, as a proxy for storage dynamics	Regular updates Free (CC BY 4.0)
**Global Dam Tracker (GDAT)** *Zhang & Gu*^39^	>31,780 dams^**‡**^	Global dam locations, their catchment areas, and other attributes	Multiple attributes, incl. height, purpose, year, installed capacity	Unknown updates CC BY-NC-ND 4.0 (non-commercial, no derivatives)
**Open Street Map** www.openstreetmap.org	>50,000 dams >35,000 reservoirs	Free cartographic database incl. tagged dam & reservoir features	Dam/reservoir name	Continuous updates Free (ODbL 1.0)
**IHA database** *IHA*^50^	>13,000 dam locations	Database of hydropower dams and their hydropower capacities in >150 countries	Unknown (not publicly available)	Unknown updates Not publicly available
**GDW database** ***(this paper)***	**41,145 barriers 35,295 reservoirs**	**Multiple types of barriers with and without associated reservoirs**	**>50 attributes, incl. height, purpose, year, volume, discharge**	**Regular updates** **Free (CC BY 4.0)**

Dams refer to generally larger river barriers. Underlined datasets were included in the creation of the GDW database (for more detailed information on them see Table 4). For the meaning of abbreviated dataset names, if not provided here, see the respective references.

^*^ICOLD catalogues coordinates of dams but releases only unreferenced images for some dams showing their location on Google Maps.

^†^Some reservoirs are formed by multi-polygons; and some polygons represent buffered outlines of reservoirs.

^**‡**^In total, the database includes >35,000 dams, but only 31,780 were georeferenced.

Overall, the lack of a consistent and regularly updated, fully georeferenced global dataset of river barriers and reservoirs is a major challenge for understanding the benefits, impacts and dependencies associated with these structures, tracking the status of river health at different spatial scales and over time, and measuring progress towards global social and environmental goals.

Notwithstanding the challenges outlined above, emerging regional and global efforts to catalogue instream barriers and reservoirs at enhanced spatial precision, completeness, and overall quality have made substantial progress in recent years. These efforts often rely on the inclusion of existing national datasets, which typically offer more comprehensive coverage of smaller barriers. For example, the Adaptive Management of Barriers in European Rivers (AMBER) dataset^7^ includes the location of 629,955 barriers across Europe; and the US National Inventory of Dams (NID)^18^ offers more than 90,000 point locations of dams for the United States. But the existence and maintenance of regional or national datasets is highly dependent on individual resources and priorities, and their use and distribution are often governed by restrictive licenses. Amalgamating these data to make them useful for global research is a complex and demanding task. Barriers and reservoirs differ in type and purpose, and the data used to represent them have typically been built following specific norms, often tailored to support institutional requirements rather than for general application. Simply merging existing regional datasets can thus introduce high uncertainties, major regional biases, and distortions in barrier density. As a particular problem, overlapping datasets can produce cascading duplicates of barriers in slightly different spatial locations; the resulting inflated numbers may have caused previous global assessments to overestimate the total amount of dams and their storage volumes. Therefore, researchers who need to blend multiple regional and global datasets to increase the coverage and accuracy of their results face time-consuming data harmonization tasks.

Advances in remote sensing technology and analyses seek to offer global coverage of river barriers and reservoirs down to ever smaller structures. For example, methods that classify land and water raster cells in millions of Landsat images over decades can detect changing waterbodies, such as reservoirs, on the Earth’s surface^19–23^. Combined with artificial intelligence and machine learning algorithms, this allows for certain aspects of the river barrier and reservoir identification process to be automated. However, methods to automatically detect small barriers without a reservoir, such as weirs and locks, across large spatial extents are more challenging, more prone to false positives and negatives, and are thus still being developed and refined for smaller extents (e.g., refs. ^24,25^). Moreover, a shift to entirely automated detection risks losing some important advantages of manual data collection and curation—including the documentation of key attribute information like dam name, purpose, designed storage volume, and construction year. The storage volume can, in some cases, be inferred using auxiliary information such as digital elevation models developed prior to dam construction or during different filling stages, yet remains challenging to ascertain for older reservoirs with little water level fluctuation. As a proxy for the construction year, the filling year of relatively recent reservoirs can be derived through automated methods that detect the onset of surface water appearances in remote sensing imagery (see *Methods*), but these estimation approaches cannot be applied for the majority of global barriers and reservoirs that were built before satellite data became available (~1980s).

The Global Dam Watch (GDW) initiative (https://www.globaldamwatch.org) has made it its mandate to address some of these challenges as a global consortium of academic institutions and non-government organizations with an interest to study dams and reservoirs (of all sizes and types) and their role within land and freshwater systems. As part of their goals, GDW aims to create and maintain the world’s most comprehensive and freely available global river barrier and reservoir repository, including associated analysis tools^26^. This aspiration is implemented by consolidating, harmonizing, and curating existing barrier and reservoir datasets, and their augmentation with missing structures and specific information on barrier and reservoir attributes. The globally consistent database described in this paper, hereon termed the GDW database (or GDW v1.0), is intended to serve as a foundational platform on which subsequent efforts can be built, in particular as new instream structures are automatically detected through time-series analyses of remote sensing imagery or machine learning techniques.

Several existing global datasets which were built using a variety of methods, from compiling repositories to remote sensing, machine learning and citizen science, were combined, cleaned, and harmonized, and new data were added (see *Methods* and Tables 2 and 4 for details). During various steps of data consolidation and curation, extensive manual inspections were carried out, and a variety of quality control techniques (see *Methods* and *Technical Validation*) were applied to detect potential errors or issues in the provided data, including inconsistencies in location, attribute information, or potential duplicate records. The locations of all river barriers and reservoirs were verified through manual or supervised automated processes, and the data records were updated and/or newly georeferenced as needed. This manual curation process was guided by a variety of online digital mapping resources, including Google Earth, ESRI Basemaps, and Bing maps.

The long-term goal of the GDW database is to encompass all types and sizes of anthropogenic instream barriers across rivers and their associated reservoirs. However, the initial mapping efforts of version 1.0, as presented here, prioritize larger dams that form reservoirs, as well as run-of-river barriers on larger rivers, for which more initial information was available in well-established global sources. We intentionally refrained from including more detailed national or regional datasets of barrier locations to avoid issues of spatial bias and to provide a database that is as consistent as possible across space to support global and transboundary analyses. It should be noted that the GDW database, despite its design as a consensus product, is not aiming to supersede any existing global, regional, or national barrier or reservoir datasets, nor to make them obsolete, as each of them has their own particular focus, characteristics, and purpose, which the generic design of the GDW database cannot encompass. The one exception to this is the Global Reservoir and Dam (GRanD) database^27^, which is fully embodied in the GDW database and will therefore be discontinued.

**Fig. 1:**
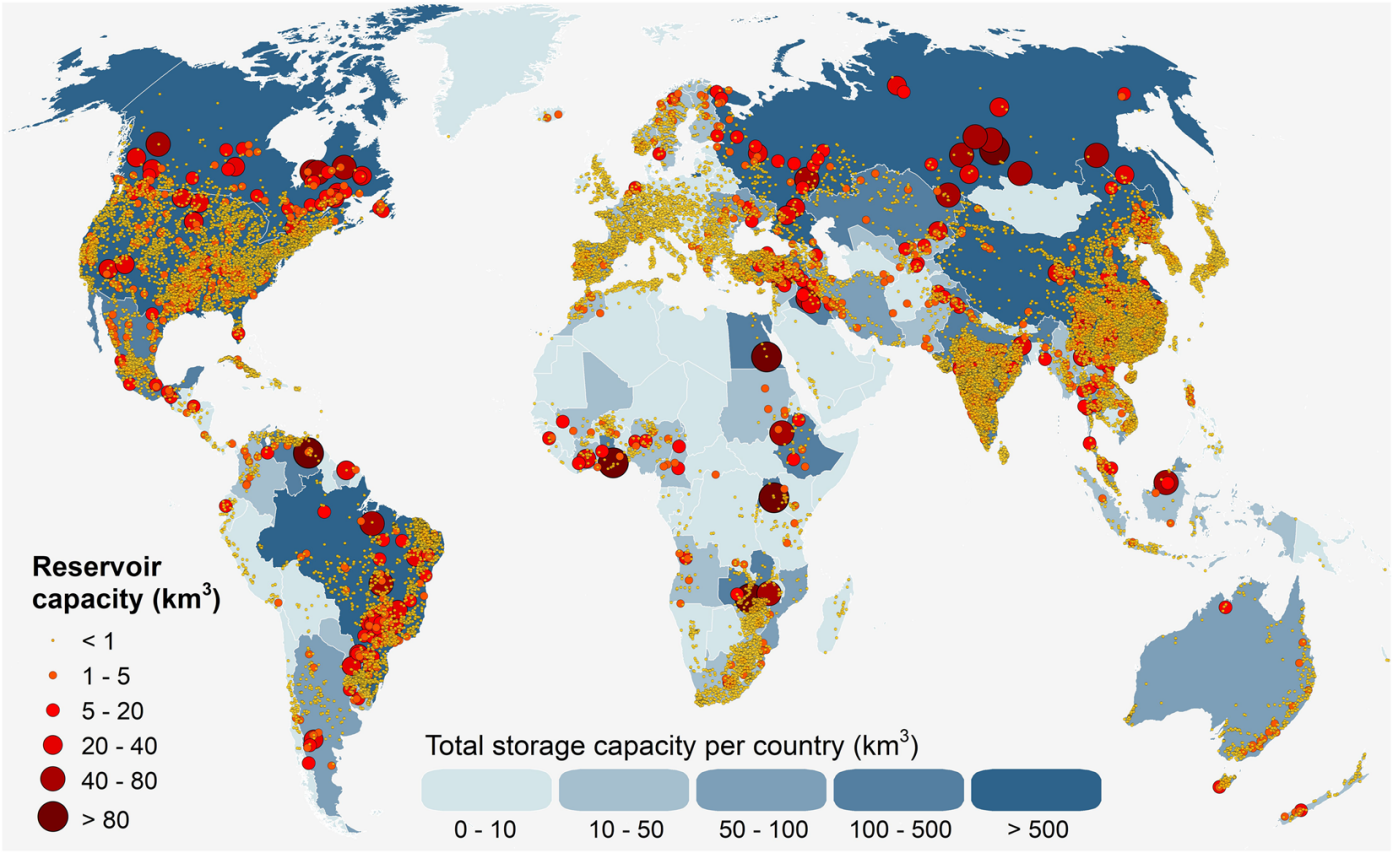
Global distribution of river barriers and reservoirs in the GDW v1.0 database. Points with reservoir capacities <1 km^3^ include river barriers that do not create a storage reservoir.

Version 1.0 of the GDW database contains point locations of 41,145 river barriers and 35,295 polygons representing their associated reservoirs (Fig. 1). It offers a wide range of barrier and reservoir attribute information, where available, and is connected to the global river network of the HydroSHEDS^28^ and RiverATLAS^29^ databases to allow for topological up- and downstream analyses and enrichment with preprocessed hydro-environmental attributes for each feature, including estimates of upstream catchment areas and discharge. GDW v1.0 is distributed under a free CC BY license.

**Fig. 2:**
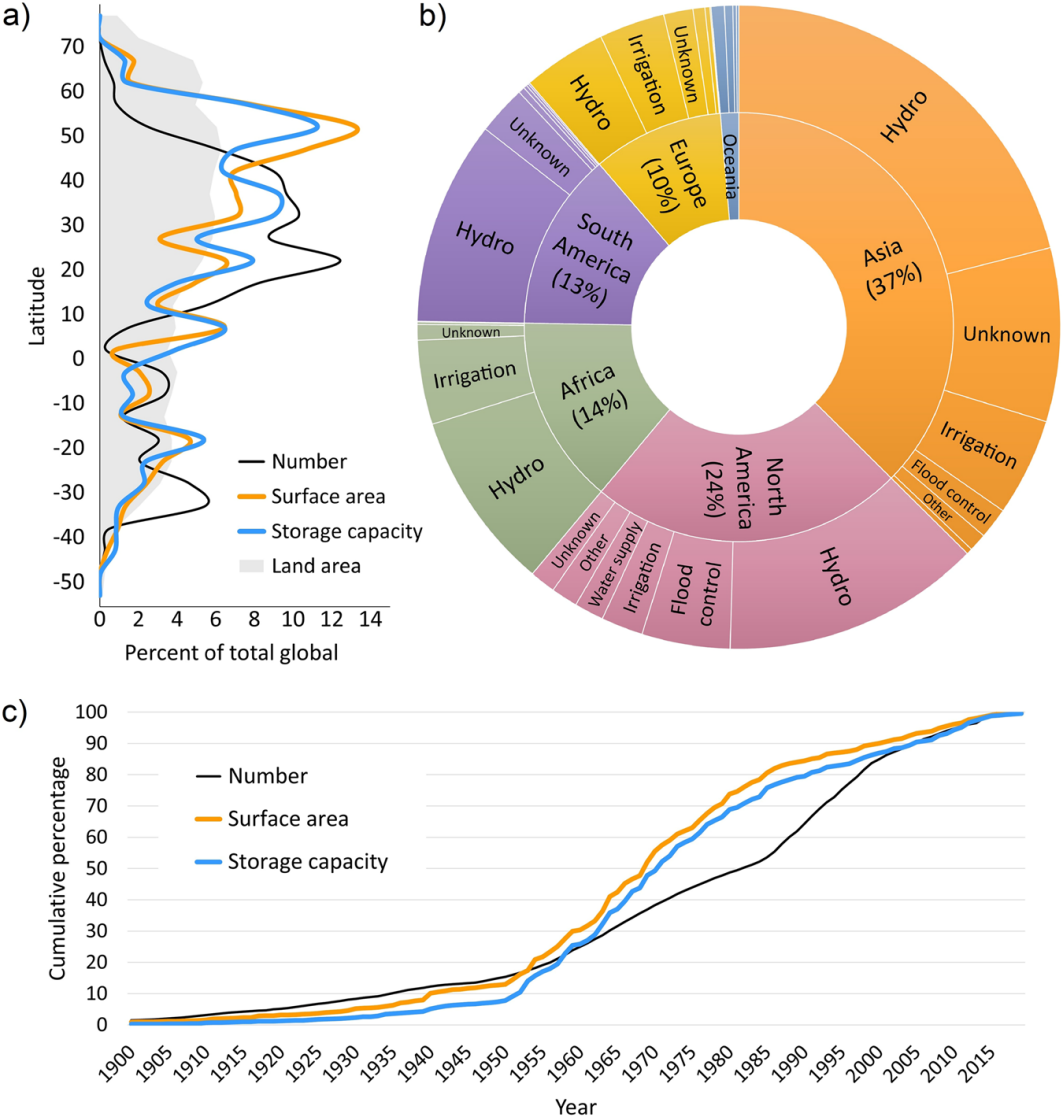
Spatial and temporal distribution of global river barriers and reservoirs. (**a**) Latitudinal distribution of river barriers and their associated reservoirs in the GDW v1.0 database; values were calculated in 5-degree latitudinal bins and drawn with smoothed lines. Grey shaded area represents distribution of global land area for reference. (**b**) Total storage capacity of all reservoirs in GDW v1.0 database by continent, indicated by their respective wedge sizes, and by primary reservoir purpose per continent, including ‘other’ and ‘unknown’ (based on main purpose, see Table 5 for more details on purpose types). (**c**) Timeline of cumulative number of river barriers and their associated total reservoir surface area and storage capacity; graphs represent only those 15,230 records (37% of entire database) which have a construction year, yet they cover 92% and 91% of surface area and storage capacity, respectively.

Figure 2 shows a) the latitudinal distribution of river barriers and reservoirs in GDW v1.0 with respect to their number, surface area, and storage capacity; b) a breakdown of the total storage volume per continent and per primary reservoir purpose; and c) a timeline of construction. In total, the GDW v1.0 database contains reservoirs with a cumulative storage capacity of 7,420 km^3^ which add a combined surface area of 304,600 km^2^ to the global inland water extent (Table 3), thus artificially expanding the global lake storage volume by about 4% and the global lake surface area by about 11%^30^. While most barriers and reservoirs have been built between 25 and 50 degrees north (Fig. 2a), including most of the United States, Europe, and China, the largest storage volumes and surface areas are reached even further north, mostly due to very large structures in Canada, Scandinavia, and Russia. Storage quantities are dominated by hydropower reservoirs across all continents, followed by different other purposes regionally, such as flood control in North America and irrigation in Africa and Europe, with unknown reservoir types contributing substantial uncertainties, in particular in Asia (Fig. 2b). The median residence time of all reservoirs (i.e., storage capacity divided by discharge) is 1.1 years, indicating that about half of the reservoirs are capable of storing the incoming flows that they receive in an entire year. The acceleration of dam construction (by number) after around 1985 (Fig. 2c) may be caused, in part, by the methods applied here to estimate construction years for reservoirs monitored by remote sensing information (i.e., after 1984). The concurrent decline in the rate of reservoir volume and area expansions, in contrast, suggests an actual slowdown in the construction of very large reservoirs. The plateauing of all curves around 2015 is likely due to incomplete records of the most recent reservoirs in GDW v1.0.

**Table 3:** Global totals of the number, surface area, and storage volume of reservoirs by size category.

Results are shown for all records in the GDW v1.0 database that have both area and volume information (excluding reservoirs that have been replaced, removed, subsumed, or destroyed), as well as extrapolated using a Pareto distribution model. Combined totals are derived by summing values from the GDW database for reservoirs ≥10 km^2^ and from the Pareto model for smaller size categories (dashed boxes). In the Pareto model, total area is derived by multiplying the number of reservoirs with the average area per category; total volume is derived using Eq. 2 (see *Methods*) to estimate the volume of the average reservoir per category and then multiplying by the number of reservoirs. For details on the Pareto model see *Methods*.

^a^Including surface areas of regulated lakes.

^b^Excluding surface areas of regulated lakes.

Table 3 provides a summary of global reservoir statistics by reservoir size category, including an extrapolation to smaller reservoirs using a Pareto distribution model (see *Methods* for details). While the Pareto model corroborates the assumption that the GDW database is comprehensive for reservoirs larger than 10 km^2^ in surface area, records for smaller reservoirs are increasingly incomplete. Using the extrapolation from the Pareto model, we estimate that there are a total of 4.4 million reservoirs worldwide exceeding 0.1 ha (0.001 km^2^) in surface area, providing a combined artificial water extent of 365,910 km^2^ (excluding regulated lakes) and a total storage volume of 8,110 km^3^. The missing reservoirs would thus expand the recorded surface area of the GDW database by ~61,000 km^2^ and its storage volume by ~700 km^3^. When applying an even lower size threshold of 0.01 ha, the number of artificial reservoirs and ponds may exceed 27 million worldwide, adding another 6,500 km^2^ in surface area and 60 km^3^ in storage volume. Despite high uncertainties in these extrapolations, they are in general accordance with independent studies that estimated about 2.9 million small reservoirs (0.0003 to 0.1 km^2^) in semi-arid regions of the world alone^31^, and about 1.8 million farm dams (0.0001-0.1 km^2^) in Australia^32^.

## Methods

### Overview

**Fig. 3:**
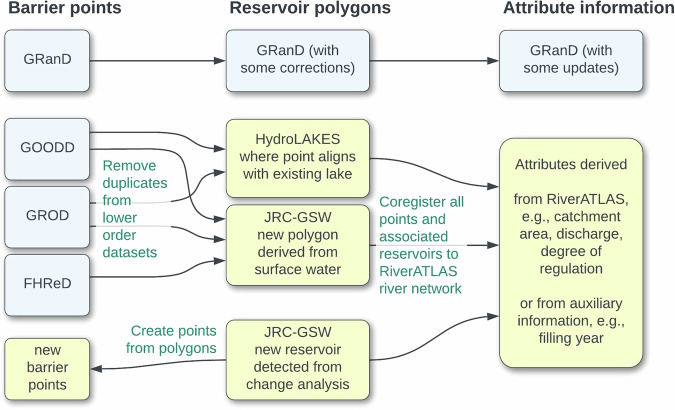
Flow diagram of the main methodological steps involved in creating, augmenting, and combining data for the creation of the GDW v1.0 database. See main text and Fig. 4 for more details. Data sources, including HydroLAKES, JRC-GSW, and RiverATLAS are described in Tables 2 and 4 and in the main text.

The foundational river barrier and reservoir database introduced here, GDW version 1.0, has been assembled by first combining the barrier and reservoir information from several complementary global source datasets: the GlObal GeOreferenced Database of Dams (GOODD)^33^; the Global Reservoir and Dam Database (GRanD)^27^, and the Global River Obstruction Database (GROD)^34^. Barrier and reservoir locations from these datasets were supplemented by the geographic coordinates of recently completed hydropower dams extracted from the Future Hydropower Reservoir and Dam database (FHReD)^35^. Reservoir polygons, where applicable, were derived from the existing GRanD reservoir dataset, the global HydroLAKES^30^ dataset, and from the remote sensing products of the Global Surface Water Explorer from the European Commission’s Joint Research Centre (JRC-GSW)^19^. As such, the GDW v1.0 database is an amalgamation of four point-based geospatial datasets (GOODD, GRanD, GROD, and FHReD), two polygon-based geospatial datasets (GRanD and HydroLAKES), and a new set of polygons and associated barrier points derived from one raster-based dataset (JRC-GSW). These data and their origins are fully described in their respective publications; an overview of their main characteristics is provided in Tables 2 and 4, and the methods by which they have been harmonized are described below and in Fig. 3. Other existing global data products (see Table 2) were omitted at this stage in order to avoid licensing issues (WRD) or partial duplication of records (GeoDAR, GWW, GDAT); instead, they served for validation purposes and may be integrated at a later stage as licenses permit.

Our main approach in combining the different datasets was to include the unique barrier and reservoir records of each source dataset while removing duplicate records across datasets. The workflow was designed to include some automated—yet supervised—steps of data amalgamation, and to visually inspect all cases of ambiguity. This approach also allowed for error detection and correction if multiple datasets showed different interpretations of the same barrier or reservoir objects (e.g., differing locations for a given barrier, or reservoir polygons with different shapes). To avoid areal overestimation, multiple overlapping reservoir polygons were not dissolved into one, but only one representative polygon was selected. To streamline the removal of duplicates and to guide the selection of representative polygons, the input datasets were prioritized and the feature from the most reliable source was chosen unless there was evidence to the contrary. GRanD (version 1.4) was selected as the highest order dataset, because it was the only dataset containing both point and associated polygon features, and it provided the largest number of attributes (which also increased its reliability due to inherent plausibility testing); as such, it was fully included in the GDW database. GOODD, with the largest number of dam and barrier points among the source datasets and with a focus on dams that form visible reservoirs, was prioritized second, followed by GROD, which was mostly used to add barriers that do not create well-defined reservoirs (i.e., weirs, locks, barrages). The global lake polygon database of HydroLAKES and the satellite-based JRC-GSW dataset were used to extract or create new reservoir polygons; in turn, polygons from JRC-GSW were also used to infer new barrier locations that were missing in the other data sources. Finally, the FHReD dataset contributed a small number of barrier points representing mostly run-of-river and newly built hydropower dams.

To enrich the attribute information and extend its versatility for subsequent applications, the GDW database has been co-registered to the global hydrographic databases of HydroSHEDS and RiverATLAS by allocating each barrier and its associated reservoir to a raster cell of the HydroSHEDS drainage direction map at a cell resolution of 15 arc-seconds (~500 m at the equator). HydroSHEDS is a mapping product that provides global-scale geospatial data on river networks and their associated catchments in a consistent format^28,36^. RiverATLAS is a postprocessed extraction of the global river network from HydroSHEDS representing all river reaches with a drainage area ≥10 km^2^ or a long-term mean discharge ≥0.1 m^3^ s^−1^, or both^29^. Each river reach is augmented by more than 50 hydro-environmental characteristics, including discharge estimates and catchment attributes. The GDW database has also been fully integrated into the global HydroLAKES dataset (version 1.1), i.e., each GDW reservoir is part of HydroLAKES, to avoid duplication or misalignment of lake outlines. The co-registration of the river barrier and reservoir information with the global river and lake network products enables the derivation of attributes such as catchment characteristics for each barrier and reservoir in the GDW database, facilitates the calculation of flow connections between the geographic features, and supports hydrologic modeling applications and the assessment of up- and downstream effects due to the instream structures.

The following sections explain the construction of the GDW database in more detail. However, as the often complex, multi-step data harmonization and fusion procedures involve information from across many “parent” datasets, reporting on every individual data manipulation step is challenging. Thus, the explanations aim to strike a reasonable balance in describing all key steps while avoiding excessive detail. Some additional information, such as a quantification of the different polygon sources, can be found in the Technical Documentation that accompanies the GDW database.

### Main data sources

The development of version 1.0 of the GDW database is primarily aimed at compiling available global barrier, dam, and reservoir information; harmonizing and curating it through both (supervised) automated and manual cross-validation, error checking, and identification of duplicate records, attribute conflicts, or mismatches; and augmenting missing information from a multitude of sources or statistical approaches. Table 4 describes the main input datasets used in this process. While the extent of all these data repositories is fully global, they show different characteristics regarding their content, comprehensiveness, and the type of attributes they provide. Differences are mostly due to the objectives of each dataset and the underpinning sources used to assemble them. For example, many of the sources for the GRanD database used a height threshold of 15 m for dams in their original collections, introducing a bias in the initial selection towards higher and larger dams.

**Table 4:** Global data sources used in the development of the GDW v1.0 database and their characteristics.

Dataset name and *reference*	Data characteristics or main purpose in creation of GDW database	Contributed objects and attributes	Number of contributed records*
**GOODD** (GlObal geOreferenced Database of Dams) *Mulligan et al*.^33^	Locations of medium to large dams that are visible on satellite imagery (dams ≥150 m long and with a reservoir ≥500 m long). River barriers were digitized by visually inspecting 1° x 1° tiles on Google Earth. Most records were compiled between 2007 and 2011 with additional updates in 2016. Most barriers contained in GOODD are associated with a reservoir, as barriers without reservoirs are more difficult to positively identify in low-resolution satellite imagery.	Barrier points	25,931
**GRanD** (Global Reservoir and Dam database) v1.4 *Lehner et al*.^27^	Large dams and reservoirs (≥0.1 km^3^); compiled from freely available data, peer-reviewed and grey literature, internet; manual inspection and validation of all records; extensive attribute information. In parallel to the creation of the GDW database, new river barriers, reservoirs, and attributes were added to the latest version 1.4 of GRanD, some errors were corrected, and new polygons were derived from JRC-GSW surface water data^19^. More details on updates can be found in the Technical Documentation of version 1.3^51^.	Barrier points and reservoir polygons; multiple attributes incl. name, year, height, purpose, reservoir volume	7,424
**FHReD** (Future Hydropower Reservoirs and Dams database) *Zarfl et al*.^35^	Hydropower dams ≥1 MW; compiled from freely available data, peer-reviewed and grey literature, internet; manual inspection and validation of all records. The original dataset focused on planned projects, from which those that were completed by 2022 were selected and transferred to the GDW database. Details of a review carried out in 2018 that identified ~400 planned hydropower projects that had subsequently been built can be found in Zarfl *et al*.^52^.	Barrier points; hydropower capacity, year of construction	205
**JRC-GSW** (Global Surface Water Explorer of European Commission’s Joint Research Centre) *Pekel et al*.^19^	Time series of surface water extents of reservoirs, mapped at a resolution of 30 m, derived from Landsat imagery. The Maximum Water Extent map of the JRC-GSW dataset was used to delineate reservoir polygons at known barrier locations. Also, new reservoirs that appeared after 1984 were automatically extracted by employing advanced big-data mining techniques and analyzing spatiotemporal dynamics derived from the JRC-GSW time series data.	Reservoir polygons; some years of construction inferred from time series of satellite imagery	1,451 newly detected reservoirs (and source of 14,015 polygons for other known barrier points)
**GROD** (Global River Obstruction Database) *Yang et al*.^34^	Instream barriers on rivers wider than 30 m at mean annual discharge, mapped through manual detection from remote sensing imagery. River barriers were digitized by visually inspecting satellite imagery at sub-meter spatial resolution against imagery of rivers identified in the Global River Widths from Landsat (GRWL) database^53^. Barriers were classified into most likely categories (incl. dams, locks, and other barrier types).	Barrier points; barrier type	6,113
**HydroLAKES** *Messager et al*.^30^	Polygon outlines for all lakes globally with a surface area ≥10 ha; these polygons were used as reservoir outlines if a barrier/dam (from GOODD, FHReD or GROD) was associated with them. The HydroLAKES database was created by compiling, correcting, and unifying several near-global and regional lake datasets.	Reservoir polygons and barrier points (lake outlets)	No new records (but source of 13,854 polygons for known barrier points)
**HydroSHEDS and RiverATLAS** *Lehner et al*.^28^*; Linke et al*.^29^	Global digital river network to which the barrier/dam locations were co-registered and from which some hydrometric attributes were derived. The river network represents all streams with a drainage area ≥10 km^2^ or a long-term mean discharge ≥0.1 m^3^ s^−1^, or both^29^ and was extracted from a gridded drainage direction map at 15 arc-second (~500 m at the equator) resolution.	Catchment area, long-term mean discharge, degree of regulation	No new records (but source of attributes for all records)

It should be noted that these collections, in turn, used underpinning information from a much wider range of sources which can be found in their respective reference papers.

^*^For original number of available records per dataset see Table 2; it is reduced here due to removal of duplicates.

All barriers and dams were geospatially referenced as point coordinates and co-registered to the global river network of HydroSHEDS and RiverATLAS (for more details see *Co-registration to a global river network* below). Where possible, the barrier/dam records were also associated with reservoir polygons. If no polygons existed in the respective input datasets, reservoir outlines were either sourced from the global HydroLAKES dataset or derived from the surface water extent maps of the JRC-GSW dataset (see *Provision and creation of new reservoir polygons* below).

While the GDW database aims to include all types of anthropogenic instream barriers, mapping efforts for version 1.0 prioritized major dams that form larger reservoirs, as well as instream barriers on larger rivers, for which more information was available. This focus on ‘larger’ structures was already inherent in the source datasets used in the compilation of the GDW database. For example, the intent of the GRanD database was to include all reservoirs with a storage capacity of more than 0.1 km^3^; the GOODD database mapped medium to large dams visible in publicly accessible remote sensing imagery; FHReD focused exclusively on proposed hydropower dams with a hydropower capacity exceeding 1 MW; and GROD mapped river barriers for rivers wider than 30 meters.

### Provision and creation of new reservoir polygons

**Fig. 4:**
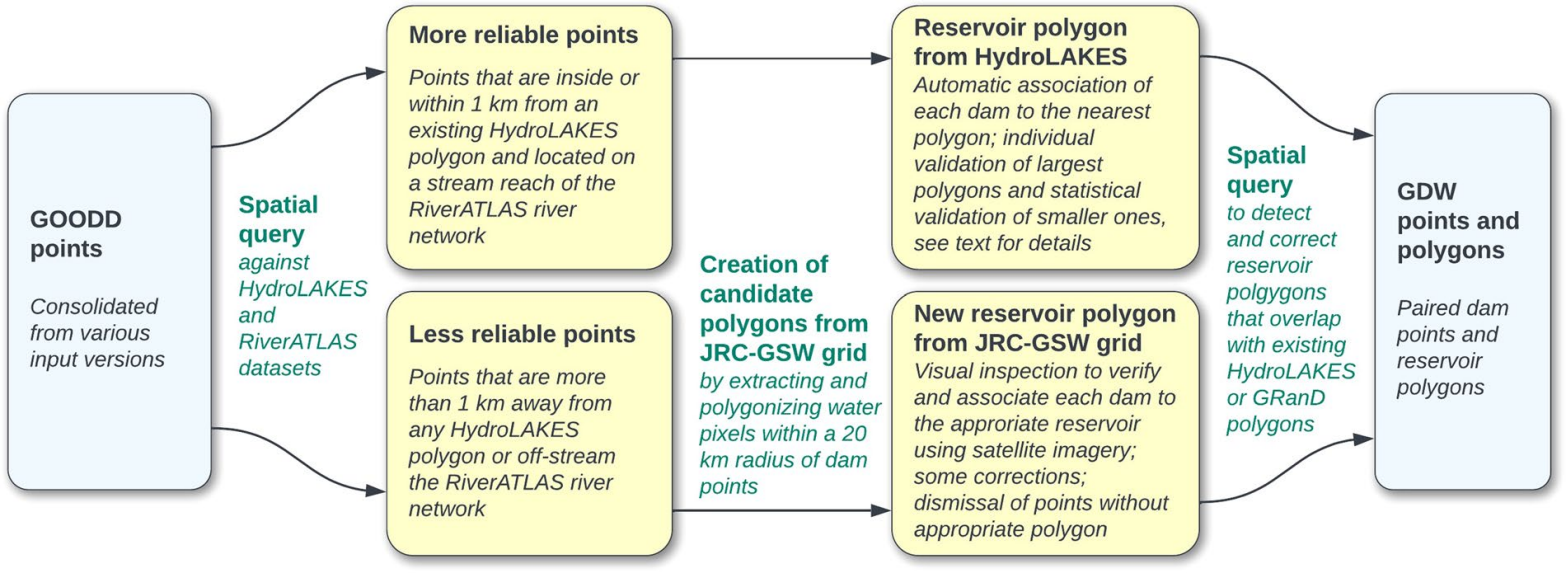
Example of reservoir creation and pairing process using barrier points from the GOODD dataset. For the JRC-GSW grid, the Maximum Water Extent map of the dataset was used. See main text for more details.

Reservoir polygons provide detailed information on the spatial extent of the reservoir that can be incorporated into modelling studies and can be used to statistically generate attribute information (e.g., reservoir volume) where none is reported. As GRanD v1.4 was the only dataset to natively provide reservoir polygons, additional candidate polygons needed to be created for barrier points that originated from the remaining datasets (see Fig. 3 for an overview). Candidate reservoir polygons were either copied from the existing HydroLAKES dataset or created from scratch using the global surface water layers of the JRC-GSW dataset (Fig. 4 outlines the process for the example of GOODD points). Choices were made between the two polygon source datasets based on characteristics of reliability and appropriateness: HydroLAKES was considered the more reliable source for reservoirs that are located on the stream network of RiverATLAS as the “lake” polygon has already been separated from the adjoining river course, a distinction that is not inherent in the surface water representation of the JRC-GSW dataset. On the other hand, reservoir outlines are typically subject to strong seasonal fluctuations due to variations in water levels; and because many polygons included in HydroLAKES are originally delineated from static remote sensing imagery taken in February 2000 (i.e., a snapshot in time), they may reflect a low-fill or dry-season state with significantly smaller-than-maximum area^30^. For that reason, off-stream reservoirs were preferentially sourced from the long-term (1984 to present) JRC-GSW dataset.

Following this prioritization, existing “lake” polygons from HydroLAKES were selected to become reservoir polygons in the GDW database if a barrier point was located inside the polygon or if they were the closest polygon to a barrier point within a distance of 1 km; only barriers that coincided with a river reach of the RiverATLAS dataset were considered in this selection (Fig. 4). For all remaining barriers without a polygon, new candidate polygons were produced from the JRC-GSW data product which is based on Landsat imagery at 30-m resolution for the time period 1984 to present^19^. First, all JRC-GSW raster cells representing the maximum surface extent of inland waters (from 1984 to 2022) were extracted within a 20-km radius of each remaining barrier point. This radius was chosen to include most of the surface area of nearby large reservoirs which can sometimes span many kilometers in length, with the understanding that a 20-km radius may also detect multiple unrelated waterbodies. Before vectorization, the JRC-GSW raster maps were modified with boundary cleaning filters to consolidate connected water surfaces and to reduce noise at reservoir edges (where the uncertainties in water classification algorithms are highest)^23,37^. The preprocessed surface water raster was then converted to polygons and postprocessed using a smoothing algorithm to slightly generalize the rasterized shorelines (i.e., to avoid sharp angles resulting from pixel edges). This procedure resulted in discrete waterbodies for the entire analyzed area (i.e., within 20 km of a barrier point). All islands smaller than 3 ha (0.03 km^2^) within a candidate reservoir polygon were dissolved into the water surface.

After the JRC-GSW candidate reservoir shorelines were created, the polygons and their associated barrier points were manually inspected and only those polygons that corresponded to a visible barrier were selected. In this consolidation process, barriers were validated and manual corrections to the reservoir polygons were applied by comparing them to ESRI Basemaps, Google maps, Yandex maps, Mapbox, JRC-GSW Occurrence Change Intensity maps, NASA Worldview imagery, and any auxiliary documents pertaining to each barrier and reservoir. In particular, adjustments were made, mostly by visual image interpretation, to isolate the reservoir from inflowing rivers, or to merge multiple pools which were falsely separated by a bridge or due to a narrow channel.

Furthermore, after employing advanced big data mining techniques and analyzing the spatiotemporal dynamics of surface waterbodies from the JRC-GSW time series data, a total of 1,451 new reservoirs filled after 1984 were identified (Fig. 3). The corresponding reservoir surfaces were extracted and vectorized into polygons. To achieve this, a growing-region algorithm, based on JRC-GSW layers and elevation data, was applied to delineate the maximum water extent associated with each of the reservoirs. New barrier points were created and associated with these reservoirs as described in the following section.

In a postprocessing step, the new reservoir polygons derived from the JRC-GSW dataset were checked for potential overlaps with the existing HydroLAKES dataset (Fig. 4). Necessary adjustments and corrections were made to remove any overlaps, either by deleting the overlapping HydroLAKES polygon or by modifying the respective shorelines. This step also informed the creation of a new version 1.1 of the HydroLAKES dataset which is fully compatible with the GDW database. Finally, for a few exceptions (n = 48), missing reservoir polygons were manually digitized using alternative sources, e.g., if new constructions existed but were not yet visible on satellite imagery.

### Pairing of corresponding barrier (point) and reservoir (polygon) objects

Where possible, the GDW database provides paired barrier (point) and reservoir (polygon) objects which are linked via unique barrier/reservoir IDs. In a first step, the new reservoir polygons derived from the HydroLAKES or JRC-GSW datasets (see above) were paired with their respective barriers. In this step, corresponding polygons were either identified through a semi-automated ‘spatial join’ procedure (i.e., associated to barrier points that fell inside or were within a distance of 1 km from an existing candidate polygon), or by manual allocations of candidate polygons that were in close vicinity (1–5 km) of barrier locations. The two largest sets of semi-automated allocations were formed by associating a total of 13,201 GOODD points with lake polygons of HydroLAKES, and 13,151 GOODD points with polygons created from JRC-GSW data. For a validation of this procedure, including manual inspections and some corrections, see section *Technical Validation* below.

Remaining barrier points that could not be associated with a reservoir in any of the polygon datasets, yet showed a discernable barrier structure in reference remote sensing imagery, were annotated as having ‘no polygon’ in the point version of the GDW database, and no associated reservoir record exists in the polygon version. These barriers, representing mostly records from the GROD dataset, may include types that do not create obvious reservoirs, such as locks and weirs, or in some cases depict barriers with reservoirs that have not yet been filled.

In a second consolidation step, each paired reservoir was associated with a final representative barrier location, which involved replacing or adjusting some of the original point coordinates. For records derived from the GRanD database, the barrier location already existed in the original source data. For reservoir polygons added from the HydroLAKES dataset, the original barrier locations could be located anywhere within the polygon or within a distance of 1 km. To introduce consistency with the HydroLAKES data format, the original barrier locations were replaced by the existing outlet points of the HydroLAKES polygons to serve as a representative barrier location. For newly created polygons from the JRC-GSW data (or from exceptional manual digitization), the final barrier locations were derived as the raster cell (at 15 arc-second resolution) with the highest upstream flow accumulation within the reservoir polygon according to the HydroSHEDS drainage maps^28^. Like in GRanD and HydroLAKES, this procedure assumes that this raster cell is the main river fallout which can serve as a proxy for the barrier location. All barrier points were placed inside the intersection between the respective reservoir polygon and the selected raster cell. Except for very small reservoir polygons, the point was typically placed at least 80 m from the polygon boundary to ensure it will remain inside the polygon even if the data were to be reprojected. Some additional exceptions and corrections were applied during manual inspections. Note that in instances where multiple barrier points were associated with a single reservoir polygon, only one point was maintained to represent the ‘main’ barrier and information on secondary dam structures on the same reservoir was stored in the attribute table (these cases are further described in columns ‘Multi_dams’ and ‘Comments’ in Table 5).

As a result of this processing workflow, each record in the GDW database—as identified by a unique ID—typically represents a paired ‘barrier-and-reservoir object’ which is defined by both a point location and a polygon outline. The point represents the location of the barrier or dam, or the ‘main’ dam in case of multiple barriers forming a single reservoir. Furthermore, barrier objects can also be defined by a point only, representing an independent barrier or dam without a ‘traditional’ reservoir, including run-of-river hydropower stations, navigation locks, diversion barrages, check dams that only briefly create storage reservoirs during flood events, weirs and other instream control barriers, or dams under construction that do not yet have a filled reservoir.

### Identification and removal of duplicates

Linking the original records of all source datasets to the same polygon features introduced a clear relationship between reservoirs and their associated barrier(s), which supported the identification and elimination of duplicate barriers. If dam or barrier points from multiple source datasets were associated with the same reservoir polygon, they were considered duplicates and only one consolidated record was kept in the GDW database. Where information existed that multiple dams are correctly associated with one reservoir (such as a main dam plus saddle dams), the main dam was kept as a point location and information about the additional dam structures was recorded in the attribute table.

For barrier and dam locations without reservoirs, duplicates were harder to detect. In iterative, semi-automated detection procedures, point locations were assigned the distance to their nearest neighboring point. Similar to the variable duplication exclusion radii applied by Belletti *et al*.^7^, all points closer than 2 km from another point or reservoir polygon were flagged and manually inspected as to whether they resembled the same object.

### Co-registration to a global river network

To enable follow-on assessments that require river network topology, such as up- and downstream analyses, each barrier was co-registered to the global digital river network of the HydroSHEDS^28^ and the related RiverATLAS^29^ databases. We chose this river network over others as it is widely used^38^ and its associated datasets provide a rich set of hydro-environmental attributes that can be utilized to derive barrier and reservoir characteristics. For all records represented by a barrier only (i.e., without an associated reservoir), the points were manually allocated to the nearest ‘topologically correct’ raster cell in the HydroSHEDS drainage map at a resolution of 15 arc-seconds (~500 m at the equator). In other words, each barrier was moved to the respective river mainstem or tributary cell that it is located on. This process was guided by remote sensing imagery (mostly Google Earth, ESRI Basemaps, and Bing maps). For records with a reservoir polygon, the reservoir’s outlet point was used as a proxy for its barrier location (see *Pairing of corresponding barrier and reservoir objects* above), which by default is located inside the raster cell that represents the main river draining the reservoir.

As the RiverATLAS dataset is directly extracted from the HydroSHEDS drainage network, the co-registered barrier locations also correspond to the river segments of RiverATLAS, thus facilitating a direct transfer of the hydro-environmental information offered in this dataset. It is critical to note, however, that the original allocation of barrier points to raster cells (rather than to line segments) enables the distinction of barriers that are not located on a mapped stream segment (see Table 4 for mapping thresholds used in the stream delineation of RiverATLAS) but instead are situated in a cell that represents a minor tributary or an off-stream location. This detailed information is essential for river network analyses and provides an important advantage over automated “snapping” approaches, such as applied in the GDAT database^39^, which can falsely co-register barriers that in reality are located on minor tributaries to the nearest mapped and therefore larger river, potentially causing incorrect catchment considerations and an erroneous overestimation of river fragmentation issues.

Although visual inspections showed good spatial correspondence between the barrier points, reservoir polygons, and the river network of HydroSHEDS and RiverATLAS, spatial offsets and uncertainties in the range of 500 m are inherent in the river delineations due to the applied raster cell resolution. Therefore, the representative barrier location on the river network is only an approximation of the true dam location. For the 6,113 barrier locations sourced from the GROD dataset, which is considered the most spatially accurate barrier dataset used here, the coordinates of both the original barrier location and the representative location on the river network were recorded in the attribute table.

### Derivation of general barrier/dam and reservoir attribute information

During the creation of the GDW database, we aimed to identify and utilize all reliable sources of attribute information available. As a foundational step, the broad range of dam and reservoir information from the GRanD database was fully transferred. Other source datasets offered only specific information, such as hydropower capacity in the FHReD dataset. Where available, reported information from these sources was integrated into the GDW database. Additional attributes were inserted from alternative sources, including regional and national datasets (see attribute column ‘URL’ in the GDW database for links to such sources, as well as https://www.globaldamwatch.org/directory for a range of national and regional datasets that we drew from). For instance, available dam and reservoir characteristics were added from the US National Inventory of Dams (NID)^18^ through a spatial join to the nearest reservoir polygon (up to a distance limit of 500 m).

Furthermore, the linkage of the GDW records with the RiverATLAS dataset^29^ allowed for the derivation of additional attributes, in particular catchment area and long-term mean discharge. The discharge values provided by RiverATLAS are based on downscaled runoff estimates from the global hydrological model WaterGAP^40^ for the period 1971–2000 and were also used to calculate the ‘Degree of Regulation (DOR)’ index for every reservoir (see Table 5). Elevation values in the GDW database were derived from the EarthEnv-DEM90 digital elevation model^41^ which was also used in HydroLAKES.

### Estimating missing reservoir volumes

During the development of the GDW database, two regression models were derived and applied to complete missing information on reservoir volumes, following the approach by Lehner *et al*.^27^:

1 $${\rm{V}}={0.553({\rm{A}}\cdot {\rm{h}})}^{0.941}$$

2 $${\rm{V}}={15.662{\rm{A}}}^{1.059}$$

where *V* = storage volume of the reservoir in 10^6^ m^3^, *A* = surface area of the reservoir in km^2^, and *h* = dam height in m.

**Fig. 5:**
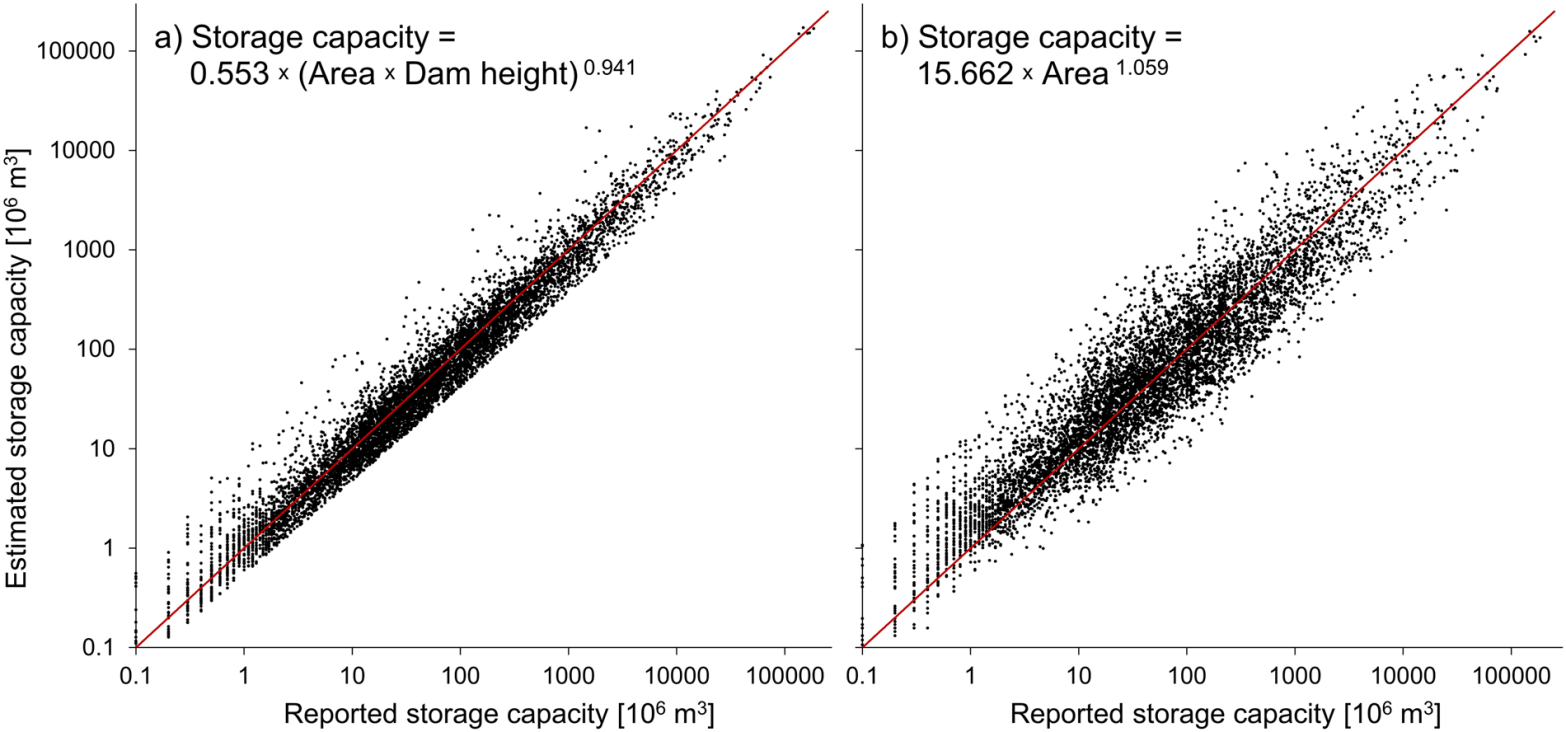
Scatter plots between reported and estimated reservoir storage volumes. Results of bias-corrected power law regression models that use (**a**) surface area and dam height as predictors (as represented by Eq. 1); and (**b**) only surface area as predictor (Eq. 2). Red lines represent the 1:1 lines.

Both equations were determined through a bias-corrected power law regression analysis of 7,348 reservoirs worldwide contained in the GDW v1.0 database which were selected based on data reliability using the following criteria: each record showed a reported reservoir volume, a reported dam height, and a calculated surface area from the associated reservoir polygon; the calculated mean depth of each reservoir (reported volume divided by polygon area) was less than the reported dam height and more than 1 m (to exclude potential lake control structures); and the quality of the record was reported as ‘Fair’ or better. Four additional records in GDW v1.0 matched these requirements but were dismissed as clear outliers after inspecting the regression scatter plots (three of these records represented an extremely large but shallow reservoir, and one had an exceptionally high dam wall). Equation 1 was used to estimate the missing storage volumes of 89 reservoirs for which both area and dam height were available (R^2^ = 0.95 for reservoirs used in the determination of the equation’s parameter settings); Eq. 2 was used to estimate the missing storage volumes of 25,504 reservoirs for which only the surface area was available (R^2^ = 0.82); see Fig. 5 for a scatterplot of both regression models. The statistical volume estimation approach was chosen over alternative methods, such as using remotely sensed surface water dynamics in combination with satellite altimetry (e.g., ref. ^42^), as it can be consistently applied for all reservoirs requiring only a surface area record. We anticipate that alternative volume estimates will be added, where available, in future versions of the GDW database.

It should be noted that Eqs. 1 and 2 were derived by relating reported storage capacities to measured polygon areas. As the polygons in many cases depict a status below full capacity, the equations may not be appropriate to estimate capacities from maximum reported areas. In instances where natural lakes are regulated by dams, such as Africa’s Lake Victoria, reported reservoir storage volumes were used; if absent, volumes were estimated from reported regulated lake depth, or by assuming a 1 m depth otherwise (such estimates were only made for 72 records).

### Extrapolating the number and size distribution of smaller reservoirs

**Fig. 6:**
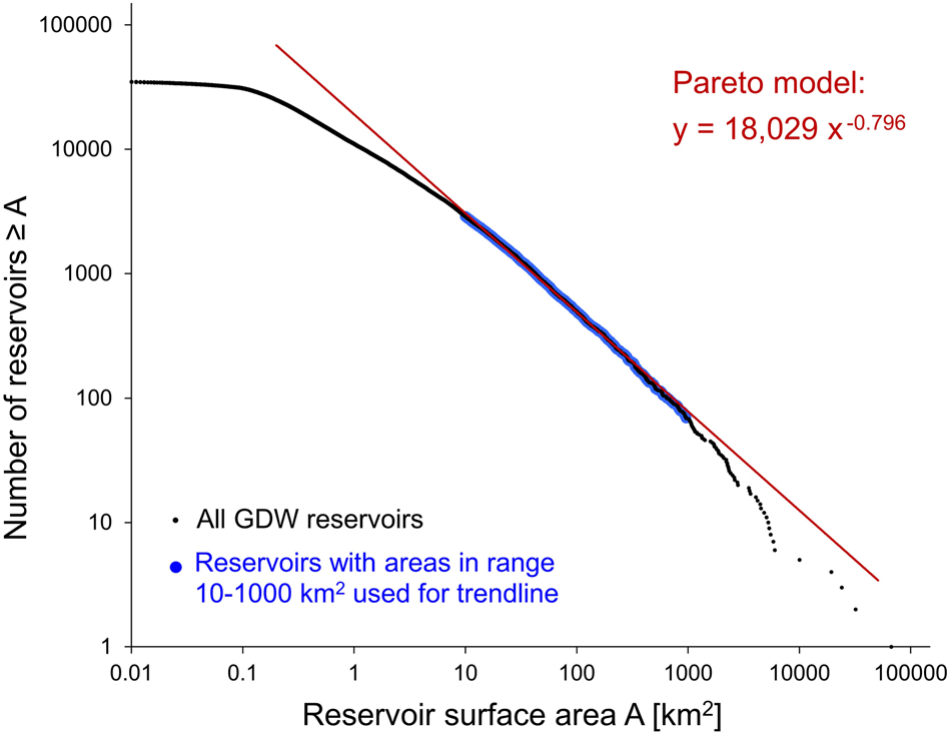
Pareto distribution model to estimate the number of reservoirs exceeding a given surface area threshold. Only reservoirs of the GDW 1.0 database in the size range between 10 and 1000 km^2^ were used to fit the Pareto model, assuming (near) completeness of these records.

To estimate the number, surface area, and storage volume of smaller reservoirs that are not recorded in the GDW database, we conducted a statistical assessment following the approach proposed by Downing *et al*.^43^ and applied in Lehner *et al*.^27^. This approach assumes that a Pareto model can be fitted to the reported reservoir distribution in the form of a power law to estimate the number of reservoirs exceeding a given surface area threshold. Using the same procedures and bias corrections as described in Lehner *et al*.^27^, yet replacing the records of the GRanD database with those of the GDW database, we derived the global reservoir distribution as:

3 $${{\rm{N}}=18,029{\rm{A}}}^{-0.796}$$

where *N* is the number of reservoirs worldwide that have an individual surface area which exceeds *A* in km^2^. A graphical visualization of this relationship is displayed in Fig. 6.

Equation 3 is the bias corrected form of the Pareto distribution which was derived considering only reservoirs in the size range of 10–1000 km^2^ (n = 2,810). Reservoirs smaller than 10 km^2^ were assumed to be increasingly incomplete in the records of GDW v1.0, while reservoirs larger than 1000 km^2^ were considered unreliable for the statistical analysis due to their increasingly random (case-specific) size. For details and equations regarding the bias correction see Lehner *et al*.^27^. Note that while the close fit of a straight line (R^2^ = 0.997) corroborates the general applicability of the approach and the underlying assumption of (near) completeness of reservoir records in the size range of 10–1000 km^2^, even a small number of missing reservoirs can affect the slope of the Pareto line and thus cause large uncertainties in the extrapolation towards smaller reservoirs. Figure 6 also reveals the onset and increasing proportion of incompleteness in the GDW v1.0 database for reservoirs smaller than 10 km^2^ and confirms the virtual absence of reservoirs below a size threshold of 0.1 km^2^. The results of this extrapolation were used to estimate average and total surface areas and storage volumes for smaller reservoir size categories (Table 3).

### Estimating the filling year for reservoirs built after 1984

For all records in the final GDW database that did not have a reported year of construction but could be associated with a reservoir polygon (n = 6,931), an estimate of the filling year was made in a two-step approach. First, a ‘candidate’ year was estimated from the JRC-GSW time series data through a heuristic statistical analysis to detect abrupt changes within the reservoir polygon from a non-water to a water surface. Second, each of these candidate years was verified (and corrected if needed) through manual inspection using timelapse remote sensing imagery built from the Landsat archive on Google Earth Engine (see https://earthengine.google.com/timelapse/). Reservoirs that were already filled before the first Landsat imagery was available in 1984 were flagged as ‘before 1985’.

While distinct changes in the timelapse sequences were observed for many records, some cases were ambiguous, either due to unclear imagery (e.g., blurred or cloud-covered scenes) or if the filling occurred close to the year 1984 (as a first visible detection of a full reservoir, say, in 1986 could also represent a reservoir that was built much longer ago, yet was empty in 1984 and 1985 due to climate fluctuations or management decisions). In all ambiguous cases (n = 839) filling years were therefore recorded as ‘before *YEAR*’ where *YEAR* refers to the first clear image of the reservoir. In a test against 111 reservoirs in the US for which years were provided in the US NID dataset, the independently made timelapse estimates were within ±5 years from the reported year for 102 records (92% of cases, including those that were correctly predicted as ‘before 1985’), within ±3 years for 98 records (88% of cases), and within ±1 year for 91 records (82% of cases). This demonstrates a good overall reliability of this estimation method.

## Data Records

The GDW v1.0 database, as presented in this manuscript, is available under a CC-BY 4.0 license at https://www.globaldamwatch.org and a copy has been deposited at the *figshare* data repository at 10.6084/m9.figshare.25988293^44^.

The GDW v1.0 database consists of two separate GIS layers: a point layer containing all representative barrier locations and their attribute information; and a polygon layer containing all corresponding reservoir outlines and their attribute information. Each barrier point lies within its corresponding reservoir polygon, thus the features and attributes of both layers can be spatially joined based on their location. Additionally, both attribute tables carry the same unique identification number for each paired barrier-and-reservoir object. Version 1.0 of the GDW database contains 41,145 barrier points and 35,295 associated reservoir polygons. That is, 5,850 barrier locations have no polygon, including navigation locks, diversion barrages, check dams that create storage only during flood events, weirs and other instream control barriers, or dams under construction that do not yet have a filled reservoir.

Both the point and polygon layer of the GDW database are offered in ESRI Geodatabase and Shapefile formats. The data are unprojected using a Geographic Coordinate System with the horizontal datum of the World Geodetic System 1984 (GCS_WGS_1984). For users without GIS software, we also included the attribute table of the barrier layer as a stand-alone text file in comma delimited UTF-8 format as part of the Shapefile package. This text file contains all GDW attribute information, and the barrier locations can be plotted using the provided x/y-coordinates.

Table 5 provides an overview of all available attribute columns available in the GDW v1.0 database, including the number of filled records. More details for each column are provided in the Technical Documentation that accompanies the data files.

**Table 5:** Attributes provided in the point (barrier) and polygon (reservoir) layer of the GDW v1.0 database.

Column title	Description *(for more information and detail, see Technical Documentation)*	Number of occurrences
GDW_ID	Unique ID for each barrier and associated reservoir	41,145
Res_name	Name of reservoir or lake (i.e., impounded waterbody)	2,098
Dam_name	Name of dam/barrier structure	10,071
Alt_name	Alternative name of reservoir or dam/barrier (including different spelling, different language)	1,806
Dam_type	Type of dam/barrier (e.g., dam, lock, lake control structure)	41,145
Lake_ctrl	Indicates reservoirs that represent a natural lake regulated by a control structure	209
River	Name of impounded river	9,501
Alt_river	Alternative name of impounded river (including different spelling, different language)	714
Main_basin	Name of main basin	2,738
Sub_basin	Name of sub-basin	721
Country	Name of country	41,145
Sec_cntry	Secondary country (indicating international dams or reservoirs associated with multiple countries)	202
Admin_unit	Name of administrative unit	41,145
Sec_admin	Secondary administrative unit (indicating dams or reservoirs associated with multiple administrative units)	4,866
Near_city	Name of nearest city	6,370
Alt_city	Alternative name of nearest city (including different spelling, different language)	302
Year_dam	Year in which the dam/barrier was built (may also refer to year of commissioning or refurbishment)	15,230
Pre_year	Estimated year before which the dam/barrier was built, as derived from remote sensing imagery	2,518
Year_src	Source of information for column ‘Year_dam’	17,749
Alt_year	Alternative year of construction (e.g., multi-year construction phase, update, secondary dam construction)	805
Rem_year	Year in which the dam/barrier was removed, replaced, subsumed, or destroyed	10
Timeline	Indicates status or change of a barrier/reservoir over time (e.g., removed, subsumed, under construction)	70
Year_txt	Summary of year information in text format	41,145
Dam_hgt_m	Height of dam/barrier in meters	9,311
Alt_hgt_m	Alternative height of dam/barrier (may indicate update or secondary dam construction)	366
Dam_len_m	Length of dam/barrier in meters	8,276
Alt_len_m	Alternative length of dam/barrier (may indicate update or secondary dam construction)	208
Area_skm	Surface area of reservoir in square kilometers; consolidated from other ‘Area’ columns	35,321
Area_poly	Surface area of associated reservoir polygon in square kilometers	35,295
Area_rep	Most reliable reported surface area of reservoir in square kilometers	7,444
Area_max	Maximum value of other reported surface areas in square kilometers	158
Area_min	Minimum value of other reported surface areas in square kilometers	289
Cap_mcm	Storage capacity of reservoir in million cubic meters; consolidated from other ‘Cap’ columns, or estimated	35,334
Cap_max	Reported ‘maximum storage capacity’ in million cubic meters	4,403
Cap_rep	Reported ‘storage capacity’ in million cubic meters; value may refer to different types of storage capacity	9,044
Cap_min	Minimum value of other reported storage capacities in million cubic meters	1,176
Depth_m	Average depth of reservoir in meters; calculated as ratio between capacity (‘Cap_mcm’) and area (‘Area_skm’)	35,321
Dis_avg_ls	Long-term (1971–2000) average discharge at dam location in liters per second; value provided by RiverATLAS	41,134
Dor_pc	Degree of Regulation (DOR) in percent; calculated as ratio between capacity (‘Cap_mcm’) and total annual discharge	35,168
Elev_masl	Elevation of reservoir surface in meters above sea level	41,134
Catch_skm	Area of upstream catchment draining into the reservoir in square kilometers; value provided by RiverATLAS	41,134
Catch_rep	Reported area of upstream catchment draining into reservoir in square kilometers	4,007
Power_mw	Hydropower capacity in MW	242
Data_info	Supporting information on certain data issues (such as source of estimated storage capacity)	27,977
Use_irri	Used for irrigation (‘Main’; ‘Major’; ‘Sec’ = Secondary use; or ‘Multi’ if multiple uses exist without a ranking)	2,669
Use_elec	Used for hydroelectricity production (‘Main’; ‘Major’; ‘Sec’; or ‘Multi’)	3,065
Use_supp	Used for water supply (‘Main’; ‘Major’; ‘Sec’; or ‘Multi’)	2,286
Use_fcon	Used for flood control (‘Main’; ‘Major’; ‘Sec’; or ‘Multi’)	2,030
Use_recr	Used for recreation (‘Main’; ‘Major’; ‘Sec’; or ‘Multi’)	2,105
Use_navi	Used for navigation (‘Main’; ‘Major’; ‘Sec’; or ‘Multi’)	322
Use_fish	Used for fisheries (‘Main’; ‘Major’; ‘Sec’; or ‘Multi’)	359
Use_pcon	Used for pollution control (‘Main’; ‘Major’; ‘Sec’; or ‘Multi’)	106
Use_live	Used for livestock water supply (‘Main’; ‘Major’; ‘Sec’; or ‘Multi’)	49
Use_othr	Used for other purposes (‘Main’; ‘Major’; ‘Sec’; or ‘Multi’); includes purposes other than those above, or mixed usage	800
Main_use	Main purpose of reservoir (incl. ‘multipurpose’ if multiple uses exist without a ranking)	8,435
Multi_dams	Indicates whether there is more than one dam associated with this reservoir (e.g., main and saddle dam)	225
Comments	Comments	964
Url	URL of related website	1,229
Quality	Quality index (verified, good, fair, poor, unreliable; for definitions see Technical Documentation)	41,145
Editor	Final data editor of entered information	41,145
Long_riv	Longitude of the point location of the dam/barrier in decimal degrees after co-registration to the HydroSHEDS drainage network	41,145
Lat_riv	Latitude of the point location of the dam/barrier in decimal degrees after co-registration to the HydroSHEDS drainage network	41,145
Long_dam	Longitude of the actual point location of the dam/barrier in decimal degrees	6,113
Lat_dam	Latitude of the actual point location of the dam/barrier in decimal degrees	6,113
Orig_src	Original dataset from which the dam/barrier or reservoir was derived	41,145
Poly_src	Original source of reservoir polygon (incl. ‘no polygon’)	41,145
GRanD_ID	Unique ID for each original record in the GRanD database (version 1.4)	7,424
Hyriv_ID	Unique ID of the associated river reach in RiverATLAS dataset (version 1.0); ID = 0 for off-stream barriers	41,106
Instream	Indicator stating whether dam/barrier is located on a river reach of RiverATLAS, or off-stream	41,145
Hylak_ID	Unique ID of the associated polygon in HydroLAKES dataset (version 1.1) and corresponding LakeATLAS dataset	31,264
Hybas_L12	Unique ID for each corresponding sub-basin at level 12 in the BasinATLAS dataset (version 1.0)	41,134

Note that the ‘number of occurrences’ refers to the point layer (41,145 barriers) and will be lower for the polygon layer (35,295 reservoirs). More details on specifics of attributes are provided in the Technical Documentation accompanying the data files.

## Technical Validation

As a composite product that has been built by harmonizing multiple existing datasets, the quality of the resulting GDW database reflects in large part the quality of its sources. Each of these source datasets has undergone its own validation (see original references as provided in Table 4). To improve data quality during the harmonization process of the GDW database, attribute information for each barrier and reservoir was cross-referenced using multiple sources to verify veracity and identify conflicts. Links to source materials were included in the respective record for reference where available. Verification efforts were performed using a combination of published information and web-based satellite and reference maps. As a result, some data errors were detected and corrected, or data gaps were filled during the consolidation and curation procedures, e.g., by consulting and adding independent sources of information, by validating whether the ratio of reservoir volume and area (i.e., the estimated average depth) is within realistic bounds, or by applying statistical approaches such as testing multiple conflicting reservoir volumes against results from estimation Eqs. 1 or 2 to identify the most plausible one. To indicate an overall estimate of reliability, a generic quality indicator (Verified, Good, Fair, Poor, Unreliable; see Table 5) was assigned to each record by the data editors. Although subjective, this indicator allows identification of records where obvious inconsistencies, uncertainties, or data gaps remain.

As part of the automated data combination steps, 13,201 dam points from the GOODD database were included in the GDW database based on their unique association to a lake polygon of the HydroLAKES dataset (i.e., they were located within the polygon or within a distance of 1 km). To verify the quality of this automated inclusion, 100 dam points were randomly selected in South America and another 100 dam points globally. These dam points were checked against Google Earth and other publicly available satellite imagery to verify whether or not a dam structure could be identified in the imagery. On visual inspection, 98 of the 100 points in South America, and 96 of the global 100 points were confirmed as dams. Of the remaining 6 points, 5 were deemed indiscernible and only one was found to be erroneous (and corrected in the GDW database after testing).

Furthermore, to ensure that the largest of the HydroLAKES polygons identified through this automated process truly corresponded to a reservoir in the landscape, the largest 1,134 polygons (by area) were each visually inspected using Google Earth and publicly available satellite imagery. This included 202 reservoirs with an estimated storage volume exceeding 100 million m^3^. Of all visually inspected polygons, 31 (2.7%) were rejected as they corresponded to a natural lake rather than a discernable reservoir with dam infrastructure.

In a second major data combination step, 13,151 dam points from the GOODD database were automatically paired with an open water polygon that was derived from the Maximum Water Extent map of the JRC-GSW dataset at the point location or within a distance of 1 km. Each of these cases was visually inspected using high-resolution satellite imagery from Google Earth, ESRI Basemaps, and other publicly available sources to verify whether it indeed corresponded to a river barrier and associated reservoir. Of the 13,151 inspected point/polygon pairings, 11,773 (89.5%) were approved, 866 (6.6%) were cases that required a revision of the point or polygon (e.g., points associated with the wrong waterbody, or polygons requiring modification), and 512 (3.9%) were rejected as no river barrier could be identified.

Despite these curation efforts, each barrier, dam, or reservoir included in the GDW database is affected by uncertainties in its respective source dataset(s). These uncertainties can relate to the location of the barrier or reservoir, or to its associated attribute information. For example, potential inconsistencies in the GRanD database include typos and order-of-magnitude errors, such as mistyped volumes by a factor of 1000; or unit mismatches (e.g., feet vs. meters). Also, in many instances the dam name is different from the reservoir name, such as Lake Mead, the largest reservoir of the US, being impounded by the Hoover Dam, making attribute associations more difficult. Another uncertainty is caused by the lack of one-to-one relationships between barriers and reservoirs: some dams, such as barrages, diversions, or run-of-river hydropower stations, may not form reservoirs; some reservoirs may have multiple dams (e.g., main and saddle dams); and some reservoirs have no dam at all, such as water stored in natural or artificial depressions. These ambiguities compound the importance of knowing from which source dataset the record was derived; this information is available as part of the GDW attributes (see Table 5).

Particular caution regarding uncertainties, missing data, and false entries is warranted when utilizing the distinction of reservoir purposes. While this information has been transferred from a variety of original datasets, including GRanD and NID, and their respective underpinning sources (e.g., FAO AQUASTAT for GRanD), or compiled from auxiliary documents including literature and online descriptions, the provided information in the GDW v1.0 database remains sketchy, error-prone, and incomplete. Major ambiguities exist for multipurpose reservoirs, and the classification into main vs. secondary purposes may be subjective. Nonetheless, given the importance of this attribute for studies requiring a coarse understanding of potential reservoir operation, which is often driven by their purpose, we chose to retain the available information in the database, even for rare types which may exhibit particularly strong regional biases (such as livestock use). To elucidate completeness of these attributes (or lack thereof), the current numbers of existing entries per reservoir type (and some additional explanations) are provided in the Technical Documentation of the GDW database. We intend to improve the quality and comprehensiveness of these attributes in future iterations, including verification through citizen scientists or through cross-referencing with alternative sources.

For additional validation and improvement purposes, attribute information listed by the International Commission on Large Dams (ICOLD) in their World Register of Dams (WRD)^45^ was consulted for some dams. Similarly, the recent publication of the GeoDAR dataset (Georeferenced global Dams And Reservoirs)^46^ offered the opportunity to detect and re-inspect some erroneous entries (~90 errors of original GRanD records were flagged through comparison with GeoDAR and subsequently corrected in the GDW database).

Finally, statistics derived from the ICOLD-WRD, GeoDAR, GWW, and GDAT datasets (see Table 2) were used to validate the relative completeness of the GDW database. ICOLD-WRD offers higher dam numbers yet a similar estimate of total global reservoir storage volume (7,334 km^3^ after removal of duplicates^46^), confirming an overall comparable coverage to the GDW database (7,420 km^3^). The recently published GeoDAR and GDAT datasets show somewhat lower total records, and no reservoir polygons in the case of GDAT. Visual comparisons of their global summary maps (not shown here) reveal very similar global patterns of spatial dam distributions as those of the GDW database (Fig. 1). Beyond the number of records and variations in regional focus, main differences between the datasets include their unique couplings with different global river networks to derive auxiliary information related to their catchments, the more extensive attribution of major dam purposes (>20,000 records) in GDAT than in the GDW database or in GeoDAR (although detailed attributes for most dams in GeoDAR can be retrieved from the proprietary ICOLD-WRD dataset through an established one-to-one relationship between GeoDAR and ICOLD-WRD), and the inclusion of smaller barrier types (locks, weirs, barrages) in the GDW database that are not available in GeoDAR or GDAT. Overall, we consider these different global datasets to be complementary to each other, each based on individual efforts and at least partially independent sources. Lastly, we overlaid the GWW reservoir polygons with those of the GDW database and found that ~25,000 records represented one-to-one matches (yet with different polygon outlines), GDW v1.0 contained ~10,000 reservoirs not included in GWW, and GWW contained ~45,000 polygons not included in GDW v1.0. Inspection of ~5,000 of the additional GWW candidate reservoirs revealed that some included multiple polygon parts belonging to the same reservoir object (thus inflating total numbers), and not every reservoir could be verified, possibly indicating uncertainties inherent in the automated derivation of GWW polygons. This finding corroborates the requirement for additional curation before including new GWW polygons into the GDW database.

## Usage Notes

The GDW database is intended for large-scale studies where globally consistent information is required. Thus, when downscaling scientific analyses on river barriers and reservoirs for regional or national assessments, data from the GDW database may serve as a starting point but should be updated and complemented by available data that suit the scale and respective purposes of such studies. More comprehensive databases may be available and should be used at the national and basin scale, including those referenced in the GDW directory (https://www.globaldamwatch.org/directory) or available via the GDW intelligence platform (https://www.globaldamwatch.org/intelligence) which brings together publicly available data at varying smaller scales for harmonization. Similarly, users should exercise caution when deriving specific global barrier or reservoir statistics. For example, given the described bias in the GDW v1.0 database towards preferentially including larger barriers, statistical interpretations of average reservoir characteristics may not be representative for reservoirs of all size categories worldwide. The focus of our database development was to create a georeferenced, curated cartographic product that can be applied in spatially explicit studies, rather than, at this stage, provide a complete record of all barriers and reservoirs globally.

While every effort has been made to quality control the entries in the GDW database as outlined above, including the provision of a simplified quality indicator (ranging from ‘verified’ to ‘unreliable’, see Table 5), it remains the user’s responsibility to judge the appropriateness of incorporating the GDW database in their respective applications. Users may need to preselect records which in turn may introduce potential biases; for example, temporal selections are more uncertain pre-1985 as remote sensing imagery only supported the estimation of filling years after 1984. Users should consider that some attributes are prone to exhibit more uncertainties than others, such as a high level of ambiguity and incompleteness for reservoir purposes (see *Technical Validation*) vs. relatively robust derivatives of certain physical parameters, such as elevation or catchment size. Additional choices can be made for certain attributes, including cases in which different values or minimum-maximum ranges are recorded for a reservoir (e.g., surface area, storage capacity, year of construction; see Table 5). Of particular importance may be the choice of whether to include or exclude reservoirs that are flagged in the GDW database as ‘regulated lakes’ because this information can have profound implications on certain applications. For example, regulated lakes should typically be excluded from assessments that account for new surface water areas stemming from reservoir construction, an issue that has often been overlooked in past analyses.

The reservoir polygons of the GDW database have been fully integrated into the HydroLAKES dataset (version 1.1), i.e., there are no overlaps or inconsistencies between the respective lake and reservoir polygon datasets. Also, all barrier points of the GDW database have been co-registered to the global digital river network of the HydroSHEDS and RiverATLAS databases via their x/y coordinates (which permits spatial joins) and by providing the corresponding ID of the associated river reach in RiverATLAS (see Table 5). These complementary data products support the derivation of additional information for the barriers and reservoirs of the GDW database (such as by transferring hydro-environmental catchment properties from RiverATLAS) and allow for versatile applications of the GDW database within the existing data frameworks of HydroSHEDS, RiverATLAS, and HydroLAKES. Barriers that are not directly located on a reach of the river network, but are located in the associated reach catchment, are distinguished and can thus be treated accordingly as ‘off-stream’ in river network analyses.

## Data Availability

All data assembly and quality-control steps were performed using sequential procedures within standard Geographic Information System (GIS) and statistical software, and no custom code was generated to automize these procedures.
